# Risk of non‐colorectal cancer‐related death in elderly patients with the disease: A comparison of five preoperative risk assessment indices

**DOI:** 10.1002/cam4.5052

**Published:** 2022-07-24

**Authors:** Kohei Yasui, Dai Shida, Yuka Ahiko, Yasuyuki Takamizawa, Konosuke Moritani, Shunsuke Tsukamoto, Yukihide Kanemitsu

**Affiliations:** ^1^ Department of Colorectal Surgery National Cancer Center Hospital Tokyo Japan; ^2^ Division of Frontier Surgery, The Institute of Medical Science The University of Tokyo Tokyo Japan

**Keywords:** cancer‐specific death, colorectal cancer, CONUT score, elderly patients, non‐cancer‐related death

## Abstract

**Background:**

A considerable number of elderly patients with colorectal cancer (CRC) die of non‐CRC‐related causes. The Controlling Nutritional Status (CONUT) score, American Society of Anesthesiologists Physical Status classification, Charlson Comorbidity Index, National Institute on Aging, and National Cancer Institute Comorbidity Index, and Adult Comorbidity Evaluation‐27 score are all known predictors of survival in patients with CRC. However, the utility of these indices for predicting non‐CRC‐related death in elderly CRC patients is not known.

**Methods:**

The study population comprised 364 patients aged 80 years or more who received curative resection for stage I–III CRC between 2000 and 2016. The association of each index with non‐CRC‐related death was compared by competing‐risks analysis such as the cumulative incidence function and proportional subdistribution hazards regression analysis as well as time‐dependent receiver‐operating characteristic (ROC) analysis.

**Results:**

There were 85 deaths (40 CRC‐related and 45 non‐CRC‐related) during a median observation period of 53.2 months. Cumulative incidence function analysis identified CONUT score as the most suitable for risk stratification for non‐CRC‐related death. In proportional subdistribution hazards regression, risk of non‐CRC‐related death increased significantly as CONUT score worsened (2/3/4 vs. 0/1, hazard ratio 1.73, 95% confidence interval [CI] 0.91–3.15; ≥5 vs. 2/3/4, hazard ratio 2.71, 95% CI 1.08–6.81). Time‐dependent ROC curve analysis showed that CONUT score were consistently superior to other indices during the 5‐year observation period.

**Conclusions:**

The majority of deaths in elderly patients with CRC were not CRC‐related. CONUT score was the most useful predictor of non‐CRC‐related death in these patients.

## Graphical abstract

More than half of elderly patients who undergo curative resection for colorectal cancer die of other diseases. The CONUT score was the most useful predictor of non‐CRC‐related death in these patients.

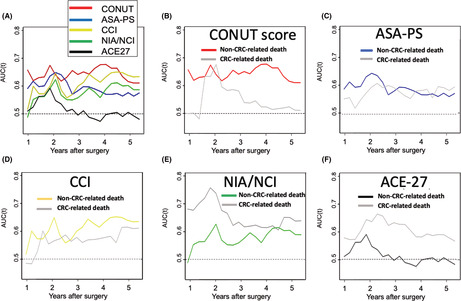

## 1 INTRODUCTION

Colorectal cancer (CRC) is primarily a disease of the elderly and is a major cause of mortality. In Japan, the incidence of CRC in the elderly population has been increasing in recent years, and now more than 25% of patients are over 80 years old.^1^ In general, postoperative morbidity and mortality rates are higher in elderly patients than those younger because of several common comorbidities and impaired functional status.^2^, ^3^ Furthermore, there is a considerable number of non‐CRC‐related deaths in the elderly, and their overall‐survival (OS) postoperatively is poorer than in younger patients.^2^, ^4^, ^5^ Moreover, no difference in cancer‐specific‐survival (CSS) after curative resection for CRC has been found between patients younger than 75 years and those aged 75 years or older.^5^, ^6^ Well‐selected elderly patients with cancer who undergo an appropriate general assessment can benefit from treatment as much as younger patients.^5^, ^7^, ^8^ Therefore, evaluation of the risk of non‐CRC‐related death is critical in determining treatment policy, preoperative management, and whether to perform normal surgery or limited surgery.

Various indices have been used to assess the general preoperative condition of patients. The Controlled Nutritional Status (CONUT) score has attracted recent attention as an index for assessing the overall condition of cancer patients from the perspective of nutrition and immunology.^9^ CONUT score is constructed with values for peripheral lymphocyte counts, serum albumin level and total cholesterol level, and offers an easy, cost‐effective method to comprehensively assess patient immune and nutritional status in conjunction with routine blood tests.^10^ CONUT score has been reported to predict OS and CSS in CRC patients.^9^, ^10^, ^11^ On the other hand, American Society of Anesthesiologists Physical Status classification (ASA‐PS) is used to stratify patients according to overall severity of illness,^12^ and Charlson Comorbidity Index (CCI),^13^ National Institute on Aging and National Cancer Institute Comorbidity Index (NIA/NCI),^14^ and Adult Comorbidity Evaluation‐27 (ACE‐27)^15^ are well‐known tools for assessing comorbidities. ASA‐PS and CCI have been reported to predict postoperative complications in CRC patients and are associated with 1‐year mortality rate in elderly CRC patients.^16^, ^17^, ^18^, ^19^, ^20^, ^21^ While CCI, NIA/NCI, and ACE‐27 are known independent prognostic factors for OS and CSS,^22^, ^23^ the relationship between general health status as defined by these indicators and the likelihood of non‐CRC‐related death in elderly CRC patients is unclear.

Colorectal cancer‐related and non‐CRC‐related deaths are competing events. In particular, when analyzing long‐term outcomes in elderly CRC patients with a high mortality risk due to other causes, it is necessary to consider events other than those related to the primary disease.^24^ Competing‐risks analysis such as the cumulative incidence function and proportional subdistribution hazards regression are appropriate for this purpose.^25^ This study aimed to evaluate the relationship between preoperative risk assessment indices and non‐CRC‐related death in older patients with CRC using competing‐risks analyses considering cancer‐specific death as a competing event against non‐CRC‐related death.

## 2 METHODS

### 2.1 Patient cohort

This retrospective study involved patients aged 80 years or more who received curative surgery for stage I–III colorectal adenocarcinoma in the Department of Colorectal Surgery at the National Cancer Center Hospital between January 2000 and December 2016. The 8th edition of the TNM staging system was used to stage the tumors.^26^ Patients with missing data necessary for the calculation of the CONUT score were excluded. The study was approved by the Institutional Review Board (IRB) of the National Cancer Center Hospital (IRB code: 2017‐437).

### 2.2 Data collection and measurement of indices of general condition

Medical records were retrospectively reviewed to collect preoperative data on age and sex, as well as the location, tumor grade, and stage of the primary tumor. CONUT scores were calculated using preoperative laboratory data for peripheral lymphocyte count, serum albumin, and total cholesterol concentration.^27^ CONUT score was calculated by summing the points for peripheral lymphocyte count (0 points for ≥1600/mm^3^, 1 point for 1200–1599/mm^3^, 2 points for 800–1199/mm^3^, and 3 points for <800/mm^3^), serum albumin level (0 points for ≥3.5 g/dl, 2 points for 3.49–3 g/dl, 4 points for 2.99–2.5 g/dl, and 6 points for <2.5 g/dl), and total cholesterol concentration level (0 points for ≥180 mg/dl, 1 point for 140–179 mg/dl, 2 points for 100–139 mg/dl, and 3 points for <100 mg/dl). All comorbidities were indexed based on ASA‐PS, CCI, NIA/NCI, and ACE‐27 scores. Using CONUT score, the participants were divided into score groups of 0/1, 2/3/4, and ≥5. Using ASA‐PS, the participants were classified according to whether the score was 1, 2, or 3. Using CCI, the participants were grouped according to whether the score was 2, 3, or ≥4. Using NIA/NCI, the participants were grouped according to whether the total number of comorbidities was 0/1, 2/3, or ≥4. Using ACE‐27, the patients were divided into none, mild, moderate, or severe comorbidity groups. Whether deaths were cancer‐specific or non‐CRC‐related was identified from the medical records. Deaths in patients without evidence of recurrence of cancer during standard postoperative follow‐up after surgery were deemed not to be CRC‐related. All confirmed cases of death in our hospital or elsewhere were investigated using medical records, telephone surveys, and letter surveys to determine the detailed causes of death, whether it was CRC‐related death or non‐CRC‐related death, and what the diagnoses were that caused the death.

### 2.3 Follow‐up surveillance

Postoperative follow‐up was performed every 3 months for the first 3 years and every 6 months thereafter, including longitudinal observation of serum carcinoembryonic antigen and carbohydrate antigen 19‐9 level. Patients were assessed by computed tomography every 6 months for 5 years and by colonoscopy 1 and 3 years after surgery as described previoussly.^28^, ^29^ Data on follow‐up were documented prospectively until an event occurred or until the study cut‐off date.

### 2.4 Statistical analysis

Survival time was defined as the interval from the date of curative surgery for CRC to the date of cancer‐specific or non‐CRC‐related death or censorship on April 30, 2020, the study cut‐off date. Cancer‐specific and non‐CRC‐related deaths were considered to be competing events. The baseline population consisted of 5005 patients aged ≤79 years who received radical surgery for CRC at the same institution from 2000 to 2016 (same period for the present study cohort). OS and CSS of the baseline population and study cohort were compared.

Patient characteristics were compared between those with CRC‐related deaths and those with non‐CRC‐related deaths using Pearson's chi‐square test. CRC‐related deaths were defined as competing events, and a cumulative incidence function was used to represent the non‐CRC‐related mortality rate in groups stratified using each index corrected. A cumulative incidence function representing CRC‐related death was also constructed. In order to compare the incidence of CRC‐related death with that of non‐CRC‐related death over time, we analyzed the cumulative incidence function in patients treated in 2000–2008 (early group) and those treated in 2009–2016 (later group). Gray's test was used to evaluate differences between groups according to the score for each index.^25^ Proportional subdistribution hazards regression was used to evaluate the relationship between each of the five preoperative risk assessment indices and non‐CRC‐related death adjusted for potential confounding covariates, including age, sex, and tumor location, tumor grade, and stage. Results are expressed as subdistribution hazard ratios (sdHRs) and 95% confidence intervals (CIs) corrected for CRC‐related death as a competing event.

Time‐dependent receiver‐operating characteristic (ROC) curves were used to compare the prognostic ability of the five indices. Time‐dependent ROC analysis incorporates a time component into the ROC analysis and evaluates the discriminatory power of continuous indices for prognosis of time‐dependent disease.^30^ A higher area under the curve (AUC) indicates that a particular variable has better discriminatory ability or prognostic accuracy. ROC curves were generated to incorporate the time dependence of disease status and each index into the ROC curve approach; the prognostic ability of each index for non‐CRC‐related death was then compared.^31^ Next, the ability of each index to predict non‐CRC‐related death and CRC‐related death was compared using Harrell's concordance index (the *C*‐index) was calculated to compare the discriminatory ability of each index. The *C*‐index is an extension of the AUC analysis to censored survival data.^32^ A larger *C*‐index value indicates better ability to predict outcomes.

All statistical analyses were performed using R version 3.6.0 (R Foundation for Statistical Computing) or JMP14 software (SAS Institute Japan Ltd.). The R package “timeROC” was used for the time‐dependent ROC analysis. The R package “cpr” was used to estimate the *C*‐index. The R package “cmprsk” was used for estimation of the cumulative incidence functions, Gray's tests, and proportional subdistribution hazards regressions. *p* < 0.05 was considered statistically significant.

## 3 RESULTS

### 3.1 Characteristics of the study cohort

A total of 367 consecutive patients aged ≥80 years who had undergone curative resection for stage I–III CRC at the National Cancer Center Hospital between 2000 and 2016 were identified. Of these, three patients lacked preoperative blood test data and were excluded. Finally, 364 patients were included in the study. The median follow‐up period was 52.4 months, and interquartile range was 31.4–64.3. Five‐year OS was 76.4% and 5‐year CSS was 86.9%. The median observation period was 65.7 months for the baseline population, with a 5‐year OS and CSS of 90.7% and 93.9%, respectively. The difference between CSS and OS, i.e., the mortality rate corresponding to the rate of non‐CRC‐related death, was 10.5% (86.9%–76.4%) in the study cohort and 3.2% (93.9%–90.7%) in the baseline population.

Table 1 shows patient characteristics. The number of patients who died during follow‐up was 85 (median 53.2 months). The cause of death was cancer‐specific in 40 patients and non‐CRC‐related in 45. The causes of non‐CRC‐related death were pneumonia (*n* = 13), cancer at another site (*n* = 11), cerebrovascular disease (*n* = 7), senility (*n* = 4), sepsis (*n* = 3), heart disease (*n* = 2), liver failure (*n* = 2), airway obstruction (*n* = 2), and pulmonary tuberculosis (*n* = 1). There was no significant difference in the distribution of age, sex, tumor location, CONUT score, or ASA‐PS, CCI, NIH/NCI, or ACE‐27 score according to whether or not death was CRC‐related. The order of the distribution of tumor grade was G1, G2, and G3 (*p* = 0.02). Patients with more advanced stages of CRC, the more CRC‐related deaths occur (*p* < 0.01). Six patients received adjuvant chemotherapy, of whom five received 5‐fluorouracil as monotherapy and one received other chemotherapy.

**TABLE 1:** Clinicopathological characteristics of patients

	Total (*n* = 364)	Cause of death		*p‐*value
		CRC‐related (*n* = 40)	Non‐CRC‐related (*n* = 45)	
Age, *n*				
<85 years	253 (70%)	25(63%)	32 (71%)	0.40
≥85 years	111 (30%)	15 (37%)	13 (29%)	
Sex, *n*				
Female	166 (46%)	16 (40%)	13 (29%)	0.28
Male	198 (54%)	24 (60%)	32 (71%)	
Site, *n*				
Colon	295 (81%)	29 (73%)	33 (73%)	0.93
Rectum	69 (19%)	11 (27%)	12 (27%)	
Tumor grade, *n*				
G1	200 (55%)	18 (45%)	25 (56%)	0.02
G2	133 (37%)	14 (35%)	19 (42%)	
G3	31 (8%)	8 (20%)	1 (2%)	
Stage, *n*				
I	109 (30%)	3 (8%)	16 (35%)	<0.01
II	132 (36%)	10 (25%)	13 (30%)	
III	123 (34%)	27 (67%)	16 (35%)	
CONUT, *n*				
0/1	215 (59%)	24 (60%)	20 (45%)	0.35
2/3/4	127 (35%)	12 (30%)	19 (42%)	
≥5	22 (6%)	4 (10%)	6 (13%)	
ASA‐PS, *n*				
1	31 (8%)	2 (5%)	1 (2%)	0.71
2	247 (68%)	25 (63%)	31 (69%)	
3	86 (24%)	13 (32%)	13 (29%)	
CCI, *n*				
2	256 (70%)	23 (58%)	24 (53%)	0.93
3	82 (23%)	13 (32%)	16 (36%)	
≥4	26 (7%)	4 (10%)	5 (11%)	
NIA/NCI, *n*				
0/1	167 (46%)	13 (33%)	15 (33%)	0.68
2/3	165 (45%)	21 (52%)	26 (58%)	
≥4	32 (9%)	6 (15%)	4 (9%)	
ACE‐27, *n*				
None/mild	0 (0%)	0 (0%)	0 (0%)	0.18
Moderate	237 (65%)	20 (50%)	29 (64%)	
Severe	127 (35%)	20 (50%)	16 (36%)	

Abbreviations: ACE‐27, Adult Comorbidity Evaluation‐27; ASA‐PS, American Society of Anesthesiologists Physical Status classification; BMI, body mass index; CCI, Charlson comorbidity index; CEA, carcinoembryonic antigen; CONUT, Controlling Nutritional Status; CRC, colorectal cancer; NIA/NCI, National Institute on Aging and National Cancer Institute Comorbidity Index.

### 3.2 Relationships between each index and the extent of surgery

Table 2 shows the relationships between five preoperative risk assessment indices and the extent of surgery (normal vs. limited). Among 364 patients, 99 (27%) underwent normal surgery (Japanese D3 lymph node dissection) and 265 (73%) underwent limited surgery with limited extent of lymph node dissection. Five‐year OS was 77.0% and 73.3% for normal and limited surgery group, respectively (*p* = 0.346), and 5‐year CSS was 86.9% and 85.7% for normal and limited surgery group, respectively (*p* = 0.929). There was no significant difference between the two groups. For CONUT 0/1, 2/3/4, and ≥5, rates of limited surgery were 75.3% (*n* = 162), 70.9% (*n* = 90), and 59.1% (*n* = 13), respectively. For ASA‐PS 1, 2, and 3, rates of limited surgery were 67.7% (*n* = 21), 72.5% (*n* = 179), and 75.6% (*n* = 65), respectively. For CCI 2, 3, and ≥4, rates of limited surgery were 69.1% (*n* = 177), 79.3% (*n* = 65), and 88.5% (*n* = 23), respectively. For NIA/NCI 0/1, 2/3, and ≥4, rates of limited surgery were 70.7% (*n* = 118), 73.9% (*n* = 122), and 78.1% (*n* = 25), respectively. For moderate and severe ACE‐27, rates of limited surgery were 70.0% (*n* = 166) and 77.9% (*n* = 99), respectively. These results suggest that rates of limited surgery increase as scores of perioperative risk assessment indices (ASA‐PS, CCI, NIA/NCI, ACE‐27) worsen, with the exception of CONUT score.

**TABLE 2:** Relationships between five indices and the extent of surgery

	Normal surgery	Limited surgery
Total, *n*	99 (27.0%)	265 (73.0%)
CONUT, *n*		
0/1	53 (24.7%)	162 (75.3%)
2/3/4	37 (29.1%)	90 (70.9%)
≥5	9 (40.9%)	13 (59.1%)
ASA‐PS, *n*		
1	10 (32.3%)	21 (67.7%)
2	68 (27.5%)	179 (72.5%)
3	21 (24.4%)	65 (75.6%)
CCI, *n*		
2	79 (30.9%)	177 (69.1%)
3	17 (20.7%)	65 (79.3%)
≥4	3 (11.5%)	23 (88.5%)
NIA/NCI, *n*		
0/1	49 (29.3%)	118 (70.7%)
2/3	43 (26.1%)	122 (73.9%)
≥4	7 (21.9%)	25 (78.1%)
ACE‐27, *n*		
None/mild	0 (0%)	0 (0%)
Moderate	71 (30.0%)	166 (70.0%)
Severe	28 (22.1%)	99 (77.9%)

Abbreviations: ACE‐27, Adult Comorbidity Evaluation‐27; ASA‐PS, American Society of Anesthesiologists Physical Status classification; CCI, Charlson comorbidity index; CONUT, Controlling Nutritional Status; NIA/NCI, National Institute on Aging and National Cancer Institute Comorbidity Index.

### 3.3 Recurrence and treatment

A total of 47 patients (13%) had recurrence, of whom 24 were treated and 23 were untreated. Seven patients received chemotherapy after recurrence and 17 underwent surgery. Regarding the proportion of patients who were treated for recurrence, no consistent trend was observed for any of the indices in each group (data not shown). Moreover, there was no significant difference in OS of patients who relapsed according to whether they received treatment or not (data not shown).

### 3.4 Cumulative incidence function curve for each index

**FIGURE 1:**
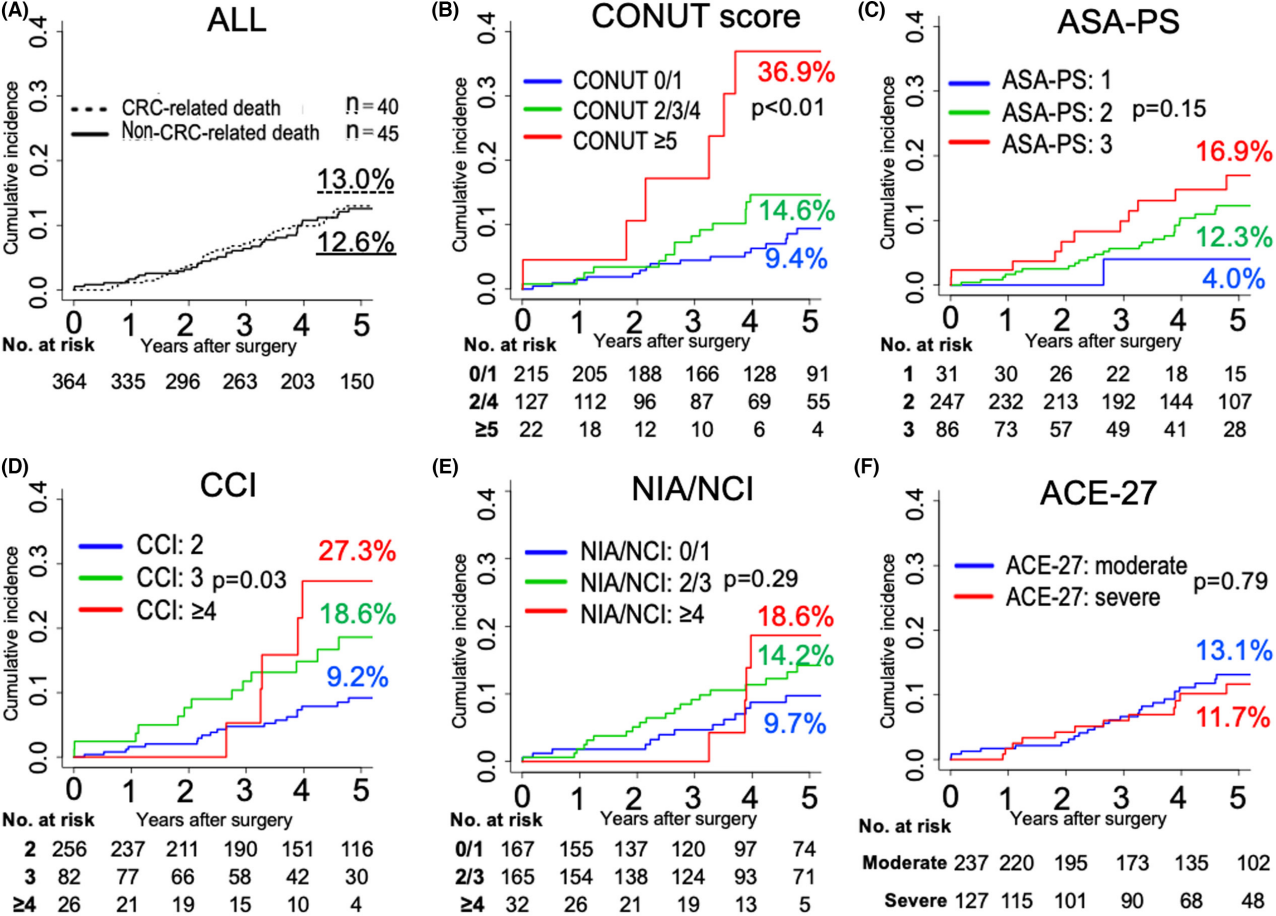
Cumulative incidence function curves for non‐CRC‐related death in elderly patients with stage I–III colorectal cancer after curative resection according to indices of general condition (*n* = 364). (A) All cancer‐specific and non‐CRC‐related deaths. (B) CONUT, (C) ASA‐PS, (D) CCI, (E) NIA/NCI, and (F) ACE‐27 scores. ACE‐27, Adult Comorbidity Evaluation‐27; ASA‐PS, American Society of Anesthesiologists Physical Status classification; CCI, Charlson Comorbidity Index; CONUT, Controlling Nutritional Status; CRC, colorectal cancer; NIA/NCI, National Institute on Aging and National Cancer Institute Comorbidity Index.

Cumulative incidence function curves for non‐CRC‐related death are shown in Figure 1. There was a similar 5‐year cumulative mortality for cancer‐specific death (13.0%) and non‐CRC‐related death (12.6%). The 5‐year cumulative mortality rates were as follows: 9.4%, 14.6%, and 36.9%, respectively, for patients with CONUT scores of 0/1, 2/3/4, and ≥5 (*p* < 0.01); 4.0%, 12.3%, and 16.9% for those with ASA‐PS scores of 1, 2, and 3 (*p* = 0.15); 9.2%, 18.6%, and 27.3% for those with CCI scores of 2, 3, and ≥4 (*p* = 0.03); 9.7%, 14.2%, and 18.6% for those with NIA/NCI scores of 0/1, 2/3, and ≥4 (*p* = 0.29); and 13.1% for those with an ACE‐27 score indicating moderate comorbidity and 11.7% for those with an ACE‐27 score indicating severe comorbidity (*p* = 0.79).

**FIGURE 2:**
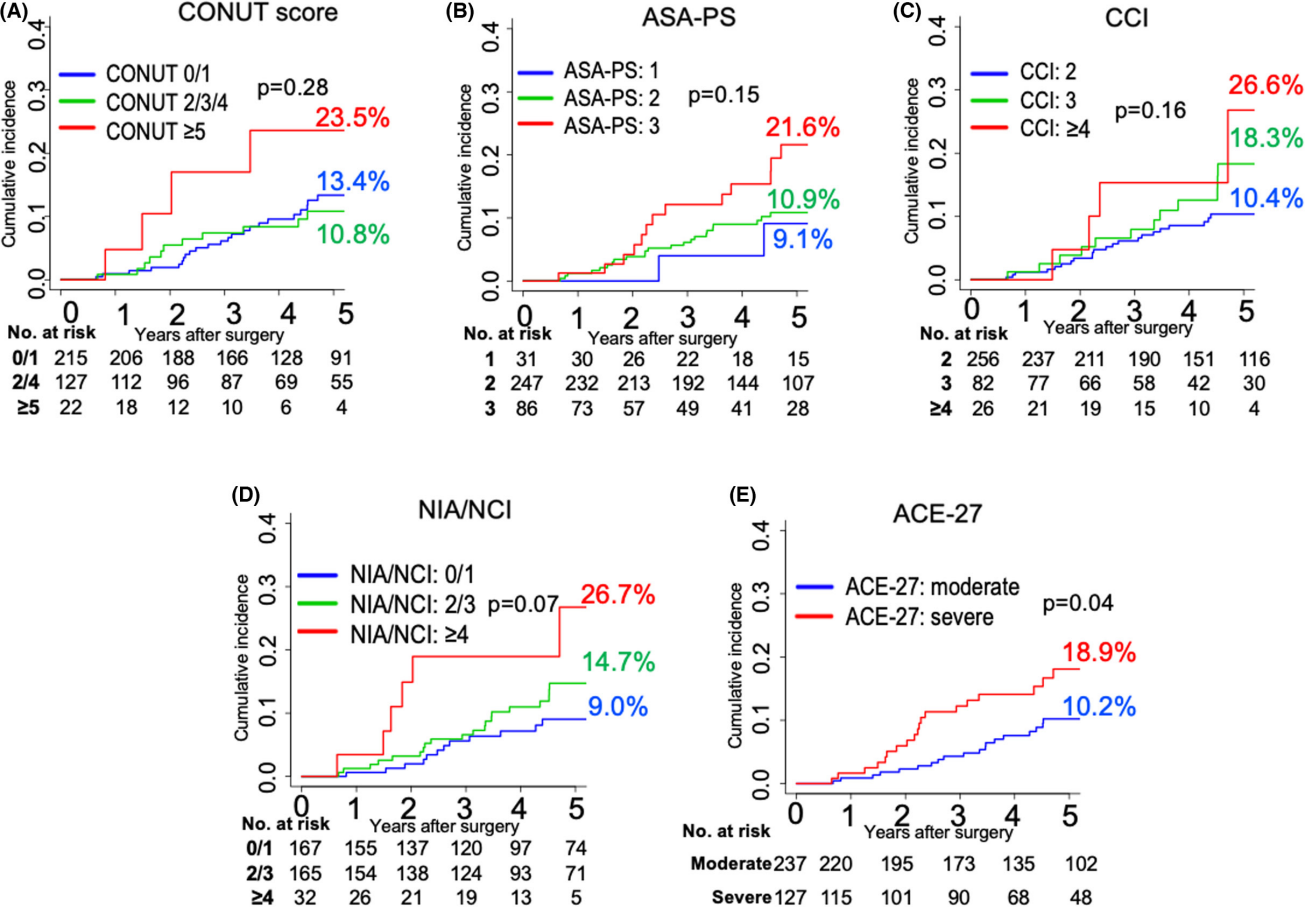
Cumulative incidence function curves for CRC‐related death in elderly patients with stage I–III colorectal cancer after curative resection according to indices of general condition (*n* = 364). (A) CONUT (B) ASA‐PS, (C) Charlson Comorbidity Index, (D) NIA/NCI, and (E) ACE‐27 scores. ASA‐PS, American Society of Anesthesiologists Physical Status classification; ACE‐27, Adult Comorbidity Evaluation‐27; CCI, Charlson Comorbidity Index; CONUT, Controlling Nutritional Status; CRC, colorectal cancer; NIA/NCI, National Institute on Aging and National Cancer Institute Comorbidity Index.

Figure 2 shows cumulative incidence function curves for CRC‐related death. There were no significant differences in the cumulative incidence of CRC‐related death in any group using CONUT score, ASA‐PS, or CCI. Furthermore, there was also no significant difference in cumulative mortality in NIA/NCI score (*p* = 0.07). However, cumulative mortality was significantly higher in the group with an ACE‐27 score indicating severe comorbidity than in the group with an ACE‐27 score indicating moderate comorbidity (*p* = 0.04).

We also analyzed the cumulative incidence function by stratifying the entire cohort according to whether they were treated in 2000–2008 (early group, *p* = 155) or in 2009–2016 (later group, *p* = 209). In the early group, the 5‐year cumulative rate was 15.1% for CRC‐related death and 17.1% for non‐CRC‐related death; the respective rates in the later group were 8.4% and 11.2% (Figure S1). The CRC‐related mortality rate was lower in the later period than in the early period, but the difference was not significant (*p* = 0.21). The non‐CRC‐related mortality rate was significantly lower in the later period (*p* < 0.01).

### 3.5 Associations between each index and non‐CRC‐related death and CRC‐related death in a competing‐risks model

A proportional subdistribution hazards regression model for non‐CRC‐related death was constructed. Table 3 shows the sdHRs for the five indices adjusted for age, sex, tumor location, and disease stage. For CONUT score, there was no statistically significant difference in sdHR for 2/3/4 versus 0/11; however, for ≥5 versus 0/1 and ≥5 versus 2/3/4, the sdHR increased significantly as the score worsened (2/3/4 vs. 0/1, HR 1.73, 95% CI 0.91–3.15, *p* = 0.08; ≥5 vs. 0/1, HR 4.43, 95% CI 1.84–10.6, *p* < 0.01; ≥5 vs. 2/3/4, HR 2.71, 95% CI 1.08–6.81, *p* = 0.03). There was no statistically significant difference in the sdHR when using CCI and ASA‐PS. However, the sdHR had trend to increase as the score worsened (For ASA‐PS, 2 vs. 1, HR 4.01, 95% CI 0.49–32.6, *p* = 0.19; 3 vs. 1, HR 6.08, 95% CI 0.71–52.0, *p* = 0.09; 3 vs. 2, HR 1.51, 95% CI 0.77–2.97, *p* = 0.23. For CCI, 3 vs. 2, HR 2.06, 95% CI 0.84–4.07, *p* = 0.10; ≥4 vs. 2, HR 2.41, 95% CI 0.99–6.13, *p* = 0.06; ≥4 vs. 3, HR 1.17, 95% CI 0.42–3.24, *p* = 0.76). Furthermore, there was no significant difference in the sdHR between groups according to NIA/NCI, or ACE‐27 score. In order to investigate the differences in results by sex, a proportional subdistribution hazards regression analysis in non‐CRC‐related deaths was performed separately for male and female.

**TABLE 3:** Association of each index and non‐CRC‐related death (*n* = 45) using a proportional subdistribution hazard model

Index	Objective variable	Reference	Adjusted^a^		
			sdHR	95% CI	*p‐*value
CONUT	2/3/4	0/1	1.73	0.91–3.15	0.08
	≥5	0/1	4.43	1.84–10.6	<0.01
	≥5	2/3/4	2.71	1.08–6.81	0.03
ASA‐PS	2	1	4.01	0.49–32.6	0.19
	3	1	6.08	0.71–52.0	0.09
	3	2	1.51	0.77–2.97	0.23
CCI	3	2	2.06	0.84–4.07	0.10
	≥4	2	2.41	0.99–6.13	0.06
	≥4	3	1.17	0.42–3.24	0.76
NIA/NCI	2/3	0/1	1.69	0.88–3.23	0.11
	≥4	0/1	1.46	0.51–4.02	0.49
	≥4	2/3	0.85	0.31–2.31	0.75
ACE‐27	Severe	Moderate	0.94	0.50–1.79	0.86

Abbreviations: ACE‐27, Adult Comorbidity Evaluation‐27; ASA‐PS, American Society of Anesthesiologists Physical Status classification; CCI, Charlson Comorbidity Index; CI, confidence interval; CONUT, Controlling Nutritional Status; CRC, colorectal cancer; NIA/NCI, National Institute on Aging and National Cancer Institute Comorbidity Index; sdHR, subdistribution hazard ratio.

^a^ Hazard ratios adjusted for age (≥85 years), sex, tumor location, tumor grade, and stage.

In the CONUT score, ASA‐PS, and CCI, the sdHR increased with worsening scores, although not significantly, and the results were similar in male and female (data not shown).

The results of proportional subdistribution hazards regression for CRC‐related death were shown in Table 4. Using CONUT score, there were no significant difference in sdHRs for the ≥5 group than in the 0/1 and 2/3/4 groups (≥5 vs. 0–1, HR 2.11, 95% CI 0.67–6.66, *p* = 0.20; ≥5 vs. 2/3, HR 2.51, 95% CI 0.76–8.36, *p* = 0.13). The 0/1 and 2/3/4 groups had a similar prognosis for CRC‐related death (2/3/4 vs. 0/1, HR 0.84, 95% CI 0.42–1.70, *p* = 0.63). Using ACE‐27, the sdHR was significantly higher in the severe group than in the moderate group (HR 1.86, 95% CI 0.98–3.51, *p* = 0.05).

**TABLE 4:** Association of each index and CRC‐related death (*n* = 40) using a proportional subdistribution hazard model

Variable	Objective variable	Reference	Adjusted^a^		
			sdHR	95%CI	*p* value
CONUT score	2/3/4	0/1	0.84	0.42–1.70	0.63
	≥5	0/1	2.11	0.67–6.66	0.20
	≥5	2/3/4	2.51	0.76–8.36	0.13
ASA‐PS	2	1	1.53	0.37–6.38	0.56
	3	1	2.81	0.64–12.4	0.17
	3	2	1.84	0.93–3.63	0.07
CCI	3	2	1.67	0.83–3.37	0.15
	≥4	2	2.15	0.68–6.81	0.19
	≥4	3	1.28	0.38–4.27	0.69
NIA/NCI	2/3	0/1	1.48	0.73–2.97	0.27
	≥4	0/1	3.08	0.98–9.60	0.05
	≥4	2/3	2.08	0.74–5.85	0.16
ACE‐27	Severe	Moderate	1.86	0.98–3.51	0.05

Abbreviations: ACE‐27, Adult Comorbidity Evaluation‐27; ASA‐PS, American Society of Anesthesiologists Physical Status classification; CCI, Charlson Comorbidity Index; CI, confidence interval; CONUT, Controlling Nutritional Status; CRC, colorectal cancer; NIA/NCI, National Institute on Aging and National Cancer Institute Comorbidity Index; sdHR, subdistribution hazard ratio.

^a^ Hazard ratios adjusted for age (≥85 years), sex, tumor location, tumor grade, and stage.

### 3.6 Time‐dependent ROC analysis and the *C*‐index

**FIGURE 3:**
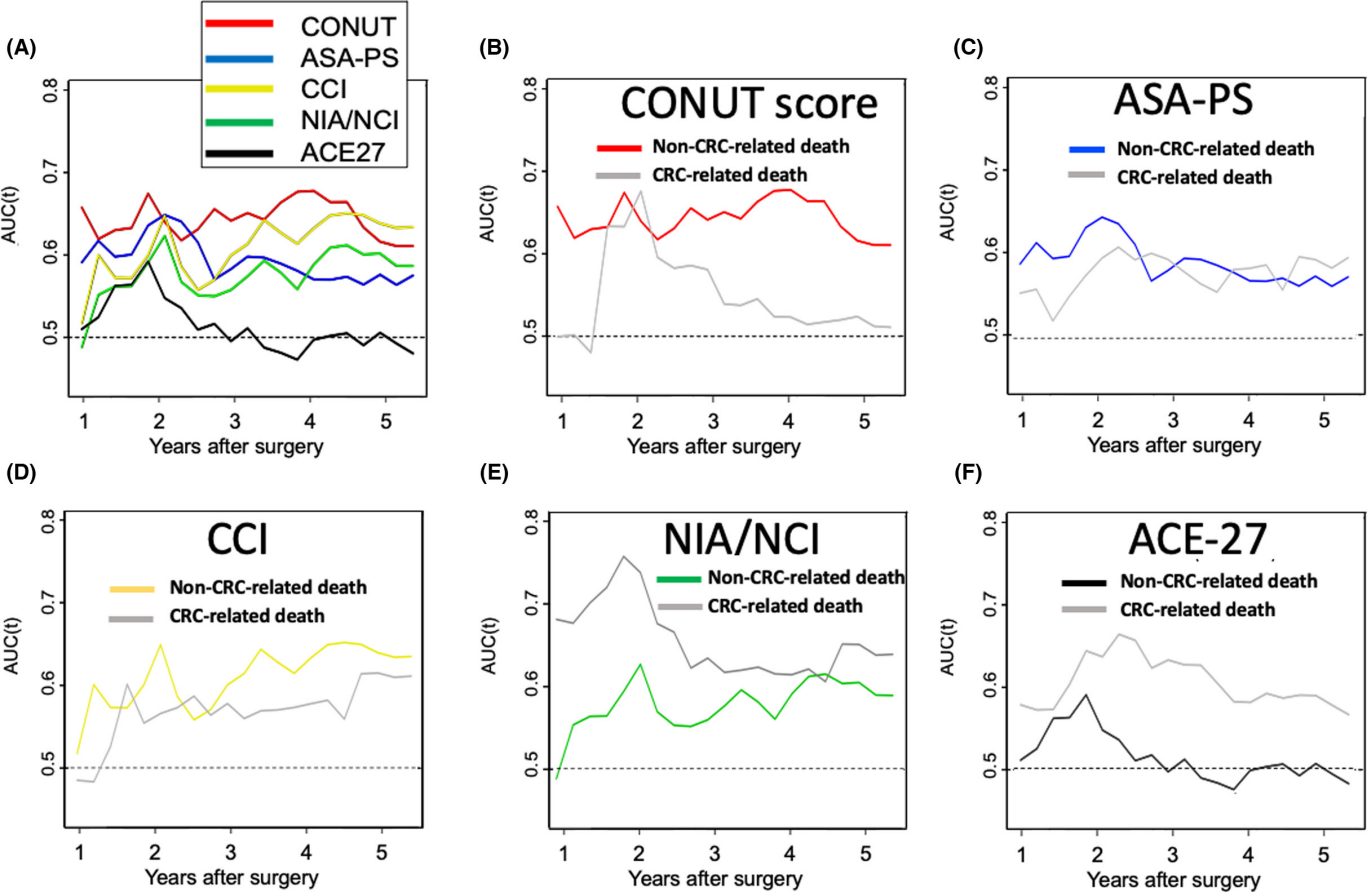
(A) Time‐dependent receiver‐operating characteristic curves showing the ability of five pre indices (A) such as CONUT, ASA‐PS, Charlson Comorbidity Index, NIA/NCI, and ACE‐27 scores to predict non‐CRC‐related death. Time‐dependent receiver‐operating characteristic curves showing the ability of preoperative (B) CONUT, (C) ASA‐PS, (D) Charlson Comorbidity Index, (E) NIA/NCI, and (F) ACE‐27 scores to predict CRC‐related death. The horizontal axis represents years after surgery. The vertical axis represents the estimated area under the curve for survival at the time of interest. ACE‐27, Adult Comorbidity Evaluation‐27; ASA‐PS, American Society of Anesthesiologists Physical Status classification; CCI, Charlson Comorbidity Index; CONUT, Controlling Nutritional Status; CRC, colorectal cancer; NIA/NCI, National Institute on Aging and National Cancer Institute Comorbidity Index.

Time‐dependent ROC curves were generated to compare the ability of each index to predict non‐CRC‐related death (Figure 3A). The AUC for CONUT score was mostly higher than that for the other indices throughout the study period, suggesting that the prognostic ability of CONUT score for non‐CRC‐related death during follow‐up was superior to that of the other indices. The *C*‐index values for predicting 5‐year non‐CRC‐related death were as follows: CONUT score, 0.632 (standard error [SE] 0.044), ASA‐PS, 0.581 (SE 0.037), CCI, 0.616 (SE 0.040), NIA/NCI, 0.582 (SE 0.042), and ACE‐27, 0.494 (SE 0.038). The *C*‐index value calculated for CONUT score was higher than that of the other indices.

Figure 3B–F shows a comparison of the time‐dependent ROC curves for CRC‐related death and those for non‐CRC‐related death for each index. Using CONUT score, the AUC for non‐CRC‐related death was mostly superior to that for CRC‐related death throughout the study period. The *C*‐index values for predicting 5‐year non‐CRC‐related death were higher than those for CRC‐related death (0.632 [SE 0.044] vs. 0.544 [SE 0.048]) when using CONUT score. For NIA/NCI and ACE‐27, the AUCs for non‐CRC‐related death were consistently superior to those for CRC‐related death throughout the study period. The *C*‐index values for predicting CRC‐related death were higher than those for predicting non‐CRC‐related death (NIA/NCI, 0.582 [SE 0.042] vs. 0.626 [SE 0.045]; ACE‐27, 0.494 [SE 0.038] vs. 0.587 [SE 0.041]).

## 4 DISCUSSION

In this study, we found that half of the deaths in patients aged ≥80 years who had undergone curative resection for stage I–III CRC were not CRC‐related. Based on the differences between OS and CSS in the study cohort and baseline population, elderly patients with CRC had a higher rate of non‐CRC‐related death (or mortality) than the baseline population (10.5% vs. 3.2%). We then performed competing‐risks analyses that considered CRC‐related (cancer‐specific) death as a competing event against non‐CRC‐related death. Using the cumulative incidence function, we showed that of the five well‐known preoperative risk assessment indices, only CONUT score and CCI were suitable for stratifying the risk of non‐CRC‐related death. Proportional subdistribution hazards regression showed that the sdHRs increased with worsening CONUT score but not with worsening CCI score. Moreover, results of time‐dependent ROC analysis suggested that the prognostic ability of CONUT score for non‐CRC‐related death was superior to that of the other four indices. Similarly, using Harrell's *C*‐index, we demonstrated that the discriminatory performance of CONUT score had the highest in all five indices. From the results of all analyses, we found that CONUT score was the most useful preoperative risk assessment index for non‐CRC‐related death in elderly patients with CRC. Moreover, both time‐dependent ROC analyses and Harrell's *C*‐index values suggested that the prognostic ability of CONUT score was higher for non‐CRC‐related death than for CRC‐related death. To our knowledge, this study is the first to consider non‐CRC‐related death and CRC‐related death as competing events when investigating the ability of commonly used prognostic indices to predict death from other causes in elderly patients with CRC.

When investigating long‐term prognosis in the elderly, planned endpoints are often not reached due to unforeseen events, namely, competing‐risk events. CRC‐related and non‐CRC‐related deaths are events that are in competition, especially in elderly patients. If a significant number of competing events occur, they may affect risk assessment of endpoint events. Traditional analysis methods, which do not consider competing risks, may overestimate the risks associated with the investigated disease. In this study, we utilized the cumulative incidence function instead of the traditional Kaplan–Meier method to represent the incidence of the endpoint of interest corrected for competing‐risk events. Moreover, instead of traditional Cox regression, we used proportional subdistribution hazards regression for the multivariate analysis, which solves the problem of competing risks in risk assessment and reflects the influence of covariates on cumulative incidence.^24^ Using competing‐risks analysis, which eliminates the problem of overestimation, CONUT score was revealed to be a useful predictor of non‐CRC‐related death in postoperative follow‐up of elderly CRC patients.

We investigated the validity of the age cutoff value in this study. We constructed Table S1, and the baseline population and the cohort of this study were divided by age into the following groups: <49, 50–59, 60–69, 70–79, and ≥80, and the number of deaths in each group was described by the cause of death. The number of CRC‐related deaths by age group was 58 (8.9%), 96 (8.3%), 106 (5.7%), 104 (7.6%), and 40 (9.5%), respectively. The number of non‐CRC‐related deaths by age group was 6 (0.9%), 21 (1.8%), 68 (3.7%), 96 (7.2%), and 45 (12.4%), respectively. There was a sharp increase in the percentage of non‐CRC‐related deaths in the ≥80‐year‐old group. The risk of non‐CRC‐related death is much higher in patients over 80 years of age than in the younger population, highlighting the importance of preoperative risk assessment for non‐CRC‐related death in this patient population. In addition, ROC curves were created using age groups and the presence of non‐CRC related deaths, and cutoff points were determined using Youden's index (AUC was 0.685). The cutoff point was over 80 years of age. Therefore, an age cutoff point of 80 years is appropriate for this study.

While the number of elderly CRC patients is increasing, their mortality rate has been decreasing in recent years.^33^ In this study, the incidence of both non‐CRC‐related and CRC‐related deaths was lower in the second half of the study period than in the first. This finding may reflect socioeconomic changes, an increasing healthy life expectancy, and improvements in the treatment of CRC and other diseases.^34^, ^35^ However, the frail elderly patients are still at higher risk of morbidity and mortality, and aggressive treatments for cancer, including surgery, chemotherapy, and radiation therapy, are usually not an option and lymph node dissection is often not performed.^36^, ^37^, ^38^

We investigated the relationships between five preoperative risk assessment indices and the extent of surgery. Among 364 patients, 99 (27%) underwent normal surgery and 265 (73%) underwent limited surgery. There were no significant differences in the respective OS and CSS. This study suggested that rates of limited surgery increase as scores of perioperative risk assessment indices (ASA‐PS, CCI, NIA/NCI, ACE‐27) worsen, with the exception of CONUT score. Therefore, limiting the extent of lymph node dissection in elderly patients with poor these scores may not affect prognosis. Thus, these indicators may be one factor in determining the extent of surgery, although large‐scale prospective studies will be needed to confirm our findings. In addition, given that the CONUT score is an index based on lymphocyte count, albumin level, and cholesterol level, each of which can be improved by improving the nutritional and immune status of patients, improvement in the CONUT score reflects improvement in nutritional and immune status, which can indirectly reduce the risk of non‐CRC‐related death. Moreover, the risk of non‐CRC‐related death as detected by the CONUT score may be directly reduced by checking the general and systemic conditions of patients and implementing interventions for those with newly discovered abnormalities and disorders. We found that the worse the score for the indices, the higher the risk of both non‐CRC‐related death and CRC‐related death, particularly for the CONUT score. Therefore, patients with poor scores should not only be aggressively treated for cancer, but they should also be carefully monitored and treated for other diseases that can cause death. In other words, these indices may serve as criteria for determining whether to take a cautious preoperative or postoperative approach. This includes determining whether surgery is overtreatment or not according to the condition of patients after the checking or interventions to patients who have high risk of non‐CRC‐related death. For instance, the indices may serve as a starting point for preoperative comprehensive geriatric assessment (CGA)‐based systemic search and problem identification. Given the considerable manpower, medical resources, time, and financial resources required to perform CGA, the CONUT score can be used as a screening tool to identify patients in need of CGA.

Several studies have reported that CONUT score is an independent prognostic factor for OS and CSS in patients with CRC.^9^, ^10^, ^11^ CONUT score assesses the peripheral lymphocyte count, serum albumin and total cholesterol, thereby reflecting immune and nutritional status. CONUT score has been shown to be associated with the prognosis of various cancers and to be an independent prognostic factor in patients with diseases such as heart failure,^39^ liver cirrhosis,^40^ ischemic stroke,^41^ and hypertension.^42^ In our study, a high point in CONUT score in elderly patients with CRC indicated poor prognosis for both CRC and other diseases. Therefore, general physical and mental health, nutritional status, and cognitive function should be rechecked in elderly patients with CRC and a high CONUT score, and consideration given to treatment of comorbidities prior to treatment for cancer. CONUT score may be one of the criteria for deciding whether patients should receive comprehensive geriatric assessment, which is a gold standard for evaluating general condition in the elderly.^3^, ^43^ CONUT score also has the advantages of being highly repeatable in clinical practice, cost‐efficient and easily measurable.^44^

This study has some limitations. First, it had a retrospective design, included patients from a single institution, and had a small sample size (only 45 patients had non‐CRC death). Second, all patients were Japanese. Since Japan is a super‐aged society with the highest rate of aging in the world, the results of the present study may not be entirely applicable to other countries. However, while Japan has the highest average life expectancy in the world, it is only 1 or 2 years longer than that of Western countries such as Spain, Italy, Switzerland, Norway, and France.^45^, ^46^ In addition, the incidence and mortality of CRC in Japan are very similar to global averages. Furthermore, the quality of medical care in Japan is on par with that of Western countries. Therefore, our results may be generalizable to other populations. Third, only patients who were deemed operable, which is a potential source of selection bias. Fourth, follow‐up may have been interrupted in some patients due to admission to a nursing home or geriatric hospital. Fifth, items such as physical functioning, socioeconomic problems, mental status, cognitive functioning, nutritional status including the presence of sarcopenia, and medication status were not assessed. These items are relevant to CGA but are not feasible to assess in all patients. Nonetheless, the present findings warrant further consideration and validation of the indices presently used to assess general condition before surgery in a larger series of elderly patients with CRC.

In conclusion, we found that half of the deaths that occurred in elderly patients with CRC in this study was non‐CRC‐related. Using competing‐risk and time‐dependent ROC analyses to compare the predictive ability of five well‐known preoperative risk assessment indices, we demonstrated that CONUT score was the best indicator of risk of non‐CRC‐related death in elderly patients with CRC.

## AUTHOR CONTRIBUTIONS

**Kohei Yasui:** Conceptualized and designed the study, conducted the analysis, interpreted the data, and prepared the first draft of the manuscript. **Dai Shida:** Conceptualized and designed the study, interpreted data, and was responsible for writing the manuscript. **Yuka Ahiko, Shunsuke Tsukamoto, Konosuke Moritani, Yasuyuki Takamizawa,** and **Yukihide Kanemitsu:** Conceptualized the study, interpreted data, and edited the manuscript.

## FUNDING INFORMATION

None to report.

## CONFLICT OF INTEREST

There is nothing to report.

## COMMERCIAL INTERESTS

None to report.

## ETHICAL APPROVAL STATEMENT

The study was approved by the Institutional Review Board (IRB) of the National Cancer Center Hospital (IRB code: 2017‐437).

## INFORMED CONSENT

This retrospective study, approved by the IRB of the National Cancer Center Hospital, was determined to be a retrospective analysis of de‐identified data, and was determined to be exempt from requiring written informed consent. IRB of the National Cancer Center Hospital approved to access the clinical/personal patient data used in our research.

## Supporting information


Figure S1

Table S1

## Data Availability

All data generated or analyzed in this study are included in the paper.
