# Supplementary material for: Risk of non‐colorectal cancer‐related death in elderly patients with the disease: A comparison of five preoperative risk assessment indices
Source: Cancer Med. 2022 Jul 24;12(3):2290–302. doi: 10.1002/cam4.5052 (PMC9939130; doi:10.1002/cam4.5052)
Supplement: Supplementary file 1 — Figure S1 [file CAM4-12-2290-s002.zip › cam45052-sup-0001-FigureS1.docx]

**SUPPLEMENTAL FIGURE LEGENDS**

Supplemental Figure. Comparison of the incidence rates for CRC-related death and non-CRC-related death between patients treated in 2000–2008 (n=155) and those treated in 2009–2016 (n=209) using the cumulative incidence function. CRC, colorectal cancer
