# Supplementary figures and images for: Risk of non‐colorectal cancer‐related death in elderly patients with the disease: A comparison of five preoperative risk assessment indices
Source: Cancer Med. 2022 Jul 24;12(3):2290–302. doi: 10.1002/cam4.5052 (PMC9939130; doi:10.1002/cam4.5052)

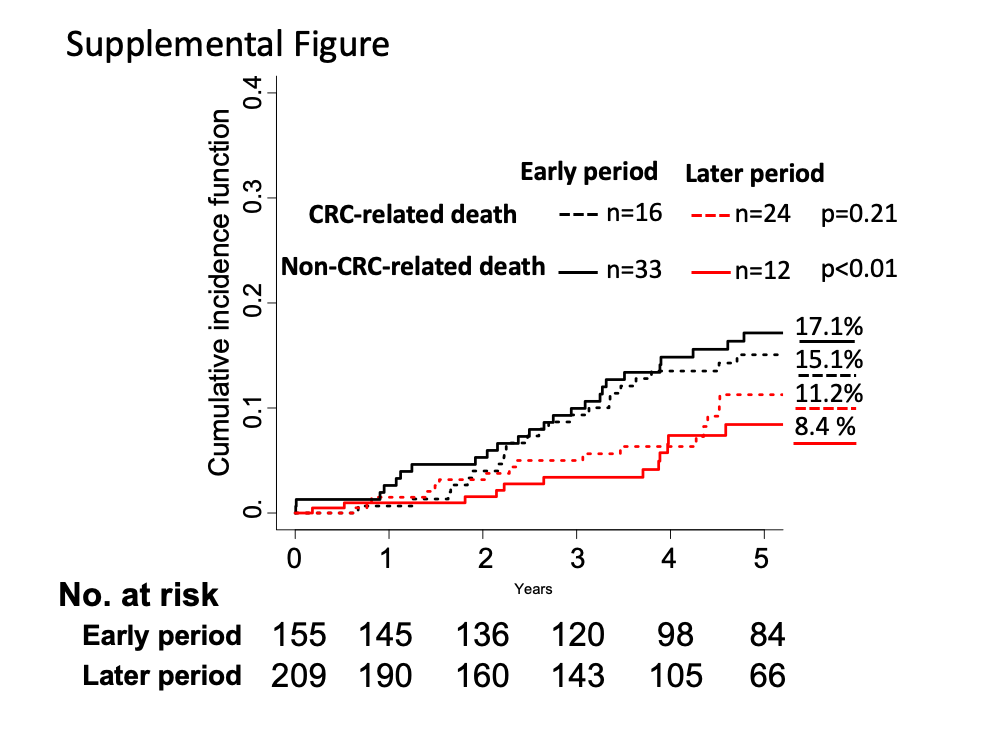

Supplement: Supplementary file 1 — Figure S1 [file CAM4-12-2290-s002.zip › CAM4_5052_20220504 KoheiYasui Supplemental Figure.tiff]
