# Supplementary material for: Risk of non‐colorectal cancer‐related death in elderly patients with the disease: A comparison of five preoperative risk assessment indices
Source: Cancer Med. 2022 Jul 24;12(3):2290–302. doi: 10.1002/cam4.5052 (PMC9939130; doi:10.1002/cam4.5052)
Supplement: Supplementary file 4 [file CAM4-12-2290-s001.docx]

Supplementary Table1. Cause of death by age group of CRC patients in our hos

Age geoup	Total (n=5372)	Cause of death	
		CRC-related (n=404)	Non-CRC-related (
<49, n	649	58 (8.9%)	6 (0.9%)
50-59, n	1157	96 (8.3%)	21 (1.8%)
60-69, n	1858	106 (5.7%)	68 (3.7%)
70-79, n	1341	104 (7.6%)	96 (7.2%)
≥80, n	367	40 (9.5%)	45 (12.4%)

[illegible]
